# Multiparameter phenotypic screening for endogenous TFEB and TFE3 translocation identifies novel chemical series modulating lysosome function

**DOI:** 10.1080/15548627.2022.2095834

**Published:** 2022-07-25

**Authors:** Phillippa J Carling, Brent J Ryan, William McGuinness, Shikha Kataria, Stewart W Humble, Stefan Milde, James A Duce, Nirav Kapadia, William J Zuercher, John B. Davis, Elena Di Daniel, Richard Wade-Martins

**Affiliations:** a Department of Physiology, Anatomy and Genetics, Oxford Parkinson’s Disease Centre, University of Oxford, Oxford, UK; b Oxford Drug Discovery Institute, Target Discovery Institute, University of Oxford, NDM Research Building, Old Road Campus, Oxford, UK; c Inherited Neurodegenerative Diseases Unit, National Institute of Neurological Disorders and Stroke, NIH, Bethesda, MD USA; d ALBORADA Drug Discovery Institute, University of Cambridge, Island Research Building, Cambridge Biomedical Campus, Cambridge; e Structural Genomics Consortium, UNC, Eshelman School of Pharmacy, University of North Carolina at Chapel Hill, Chapel Hill, NC USA

**Keywords:** High-content screening, lysosome activity, lysosome biogenesis, PKIS2, protein aggregation, TFEB

## Abstract

The accumulation of toxic protein aggregates in multiple neurodegenerative diseases is associated with defects in the macroautophagy/autophagy-lysosome pathway. The amelioration of disease phenotypes across multiple models of neurodegeneration can be achieved through modulating the master regulator of lysosome function, TFEB (transcription factor EB). Using a novel multi-parameter high-throughput screen for cytoplasmic:nuclear translocation of endogenous TFEB and the related transcription factor TFE3, we screened the Published Kinase Inhibitor Set 2 (PKIS2) library as proof of principle and to identify kinase regulators of TFEB and TFE3. Given that TFEB and TFE3 are responsive to cellular stress we have established assays for cellular toxicity and lysosomal function, critical to ensuring the identification of hit compounds with only positive effects on lysosome activity. In addition to AKT inhibitors which regulate TFEB localization, we identified a series of quinazoline-derivative compounds that induced TFEB and TFE3 translocation. A novel series of structurally-related analogs was developed, and several compounds induced TFEB and TFE3 translocation at higher potency than previously screened compounds. KINOME*scan* and cell-based KiNativ kinase profiling revealed high binding for the PRKD (protein kinase D) family of kinases, suggesting good selectivity for these compounds. We describe and utilize a cellular target-validation platform using CRISPRi knockdown and orthogonal PRKD inhibitors to demonstrate that the activity of these compounds is independent of PRKD inhibition. The more potent analogs induced subsequent upregulation of the CLEAR gene network and cleared pathological HTT protein in a cellular model of proteinopathy, demonstrating their potential to alleviate neurodegeneration-relevant phenotypes. **Abbreviations:** AD: Alzheimer disease; AK: adenylate kinase; CLEAR: coordinated lysosomal expression and regulation; CQ: chloroquine; HD: Huntington disease; PD: Parkinson disease; PKIS2: Published Kinase Inhibitor Set 2; PRKD: protein kinase D; TFEB: transcription factor EB.

## Introduction

It has been recognized that lysosomal genes have coordinated patterns of expression, which are known as the coordinated lysosomal expression and regulation (CLEAR) network. Subsequently, the TFEB (transcription factor EB) was identified as the major regulatory transcription factor which binds to a conserved element in the promoter of CLEAR genes and induces their expression [1,2]. TFEB belongs to the microphthalmia-associated transcriptional factor (MIT) family of transcription factors alongside MITF, TFE3 and TFEC [3]. TFEB and TFE3 both regulate a similar set of genes and their cellular localization is regulated in a similar manner in response to various cellular stresses [4]. While knockout of *Tfeb* in mice is embryonic lethal, knockout of *Tfe3* was initially reported to have no apparent phenotype [5,6], but subsequent studies show defects in energy metabolism via dysregulated glucose homeostasis and lipid catabolism, suggesting that despite a large degree of overlap in functionality between TFEB and TFE3, they may retain some independent functions [7,8].

The shuttling of TFEB to the nucleus responds to various environmental stimuli, such as nutrient starvation, infection, mitochondrial damage and ER stress [13; Review]. This translocation is regulated primarily by kinase signaling pathways, which ultimately result in the phosphorylation of TFEB on several serine residues to affect cellular localization. The first kinase identified as a regulator of TFEB was MTOR (within the MTORC1 complex), which phosphorylates TFEB on at least three serine residues: S122, S142 and S211 [9]. In addition to MTORC1, TFEB can be phosphorylated by MAPK1/ERK2 at S142, also resulting in cytoplasmic retention [10]. These phosphorylation sites regulate TFEB cellular localization by masking a nuclear localization signal and enhancing binding of the E3 ubiquitin ligase STUB1 to TFEB, increasing TFEB turnover via the ubiquitin proteasome [11,12]. GSK3B/GSK3β has independent phosphorylation sites at S134 and S138, which are required for the localization of TFEB to the lysosome [13].

More recently, TFEB has been shown to be phosphorylated by AKT at serine S467, in an apparently MTOR-independent manner, which can be prevented by classical AKT inhibitors and by the autophagy inducer trehalose. AKT inhibition does not alter phosphorylation at the MTOR site S211, and is able to translocate TFEB to the nucleus even in the presence of constitutively active MTORC [14].

It is likely advantageous to identify small molecule compounds that are capable of activating endogenous TFEB rather than rely on overexpression of TFEB for compound screening. To that end we have developed a high throughput screening cascade which identified compounds capable of translocation of endogenous TFEB and TFE3 to the nucleus without inducing lysosomal or cellular toxicity. Given that basic compounds can be sequestered into acidic organelles, such as lysosomes, leading to disruption of lysosomal function and inducing TFEB activation via MTORC inhibition [15–17], we included additional assays that monitor lysosomal activity and cellular toxicity. These secondary assays ensured that any targets identified are not acting via secondary toxic feedback mechanisms.

We have validated this approach using the Published Kinase Inhibitors 2 (PKIS2) compound library and identified novel chemical series, which ultimately increase lysosome function and improve protein aggregate clearance. This method can be adapted to larger, chemically diverse libraries in an effort to identify compounds or protein targets regulating TFEB and lysosome function.

## Results

### Optimization of multiparameter screening assays for TFEB and TFE3 translocation and lysosomal activity

**Figure 1.**
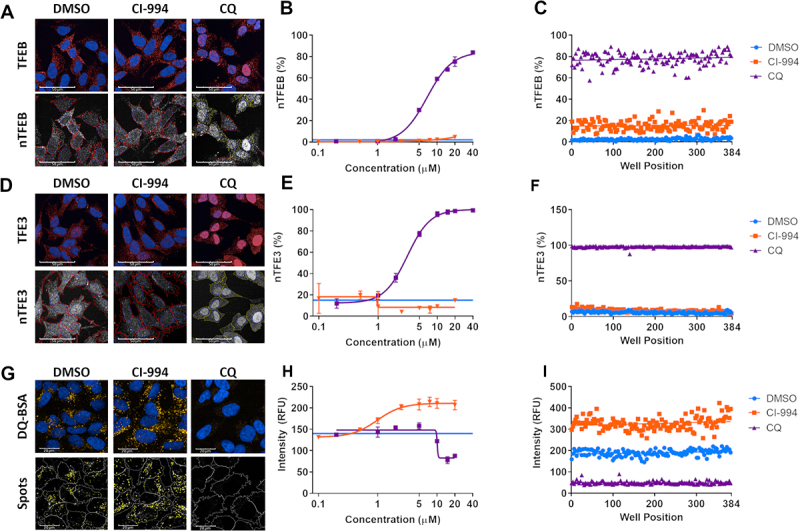
Multiparameter assays for TFEB and TFE3 translocation and lysosome activity. (A) Endogenous TFEB stained by immunocytochemistry can be seen to translocate at high levels to the nucleus upon treatment with chloroquine (CQ; 20 µM). The ratio of fluorescent signal in the cytoplasm:nucleus regions determines the percentage of cells which show nuclear translocation (nTFEB/nTFE3). Scale bars: 50 µm. (B) TFEB translocation in response to CQ is concentration-dependent and (C) can be robustly quantified across a plate in 384-well format. (**D, E, F**) Endogenous TFE3 stained by immunocytochemistry can be quantified in a similar manner to previously described for TFEB and shows the same pattern of translocation with CQ. Scale bars: 50 µm. (G) SH-SY5Y treated with DQ Red BSA reagent develop yellow fluorescent puncta under vehicle-only culture conditions, the signal of which is increased upon treatment with the HDAC inhibitor CI-994 (10 µM) and reduced by lysosomal inhibition with CQ (20 µM). The puncta are segmented and quantified to give spot count and fluorescent intensity. Scale bars: 20 µm. (H) CI-994 increases and CQ reduces lysosome count of DQ Red BSA puncta in a concentration-dependent manner. (I) The alterations in lysosome count can be robustly quantified across a 384-well plate. Images show CI-994 at 10 µM and CQ at 20 µM. n = 1. Values represent mean technical replicates ± s.d.

Rather than relying on overexpression of fluorescently-tagged TFEB and TFE3 constructs, which may alter physiological regulation, we have optimized immunocytochemistry-based assays for assessing translocation of endogenous TFEB and TFE3 using previously validated antibodies [13] confirmed in our assays to be selective (Figure S1). Lysosomal inhibition with chloroquine (CQ) induced >80% TFEB and TFE3 translocation from the cytoplasm to the nucleus in a concentration-dependent manner (Figure 1A, B, D, E) as quantified using Harmony Analysis Software (Perkin-Elmer) (TFEB: Figure S2; TFE3: Figure S3). These assays are sensitive enough to accurately detect concentration-dependent responses and are robust in a 384 well assay format (Z’ TFEB 0.7; TFE3 0.91 for DMSO to CQ comparison; Figure 1C, 1F).

In addition, we measured active proteolysis within the lysosomes using the DQ Red BSA reagent, in which fluorescent puncta are visible under standard culture conditions when the substrate is cleaved in the acidic lysosomes but are greatly reduced when cells are treated with the lysosomal inhibitor CQ, as the undegraded substrate remains quenched (Figure 1G). These DQ Red BSA-positive puncta were identified and the fluorescence intensity quantified using Harmony Analysis Software (Figure S4). Given HDAC inhibitors upregulate lysosome function [18], we tested the class I HDAC inhibitor CI-994 and found it to robustly increase DQ Red BSA puncta signal in a concentration-dependent manner (Figure 1H). The DQ Red BSA assay was also established in a 384-well plate format providing acceptable screening metrics (DQ Red BSA intensity Z’ 0.5 for DMSO to CQ comparison; Figure 1I). From this work, we selected CQ and CI-994 as positive controls for TFEB and TFE3 translocation and DQ Red BSA, respectively, to include in the subsequent screening plates.

These lysosomal assays were complemented by measuring compound cellular toxicity through counting live nuclei in microscopy images and using the ToxiLight Nondestructive Cytotoxicity BioAssay as a measure of AK (adenylate kinase) released into the media by dying cells.

### Screening of the published kinase inhibitor set 2 (PKIS2) library

The Published Kinase Inhibitor Set 2 (PKIS2) compound library comprises small molecule inhibitors of kinases. Compounds within PKIS2 have been previously profiled for inhibition of 392 wild-type human kinases (KINOME*scan* panel), with >90% showing K_d_ <1 µM against at least 1 kinase target and 357 compounds being specific to <4% of profiled kinases [19].

**Figure 2.**
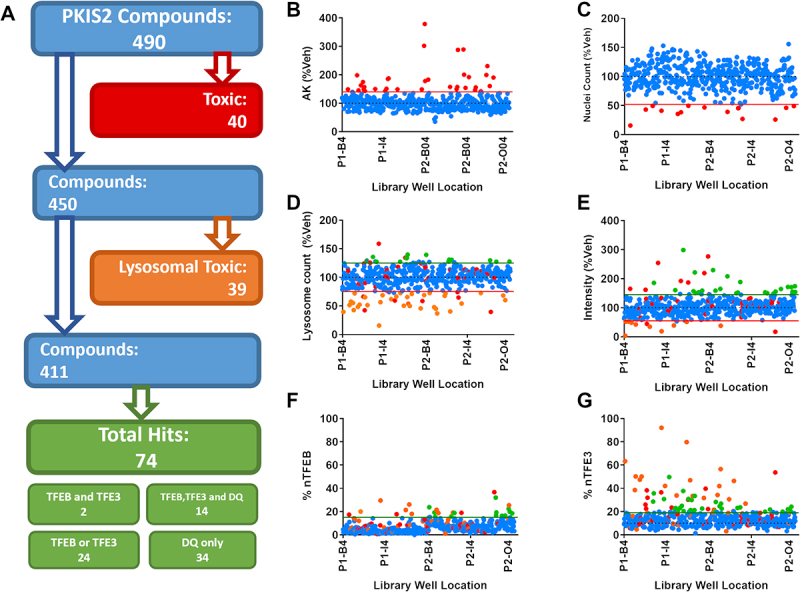
Screening the PKIS2 library. (A) The 490 PKIS2 compounds were screened at 1 µM across all assays and passed through an analysis cascade to identify nontoxic compounds which translocate TFEB and/or TFE3. (B) AK release and (C) nuclear count from immunocytochemistry images are used to assess cellular toxicity, indicated by those compounds (in red) which fall outside the 2 s.d. range of the vehicle controls (indicated by the red line). (D) Active lysosome count and (E) corrected fluorescence intensity as measured from the DQ Red BSA assay are used to assess lysosomal toxicity, indicated by those compounds (in Orange) which fall below the 2 s.d. range of the vehicle controls (indicated by the Orange line). Any which fall above the 2 s.d. range of the assay are classified as positive hits for DQ Red BSA. Remaining compounds with no associated toxicity which show nuclear translocation above the 3 s.d. range of the vehicle-only control for (F) nuclear TFEB (% nTFEB) and/or (G) nuclear TFE3 (% nTFE3) translocation are classified as positive hits. A total of 74 positive hits were identified. n = 1.

From the PKIS2 library, 490 available compounds were screened at 1 µM across the AK, lysosomal DQ Red BSA assay and TFEB and TFE3 translocation cellular assays and followed by an analysis cascade to determine positive, nontoxic hits (Figure 2A). After a 24 h treatment, 40 compounds were defined as cellular toxic as assessed by AK release (Figure 2B) and nuclear count (Figure 2C) and an additional 39 were lysosomal toxic, as defined by a decreased number of active lysosomes or DQ Red BSA intensity (Figure 2D-E). Of the 79 compounds which were cellular or lysosomal toxic, 36 (45.5%) showed positive translocation of TFEB or TFE3, highlighting the importance of our multiplexing translocation assays with measures of toxicity. Of the 411 remaining compounds which showed no toxicity, 74 compounds (15.4%) increased TFEB and/or TFE3 relocalization and/or positively regulate lysosome activity (Figure 2F-G). Of those 74 compounds, 14 increased both TFEB and TFE3 translocation and increased DQ Red BSA signal, suggesting that it may be possible to increase lysosomal function via a TFEB or TFE3-dependent mechanism. Full screening results are available in Table S1.

### Identification of chemotypes enriched for TFEB and TFE3 induction

**Figure 3.**
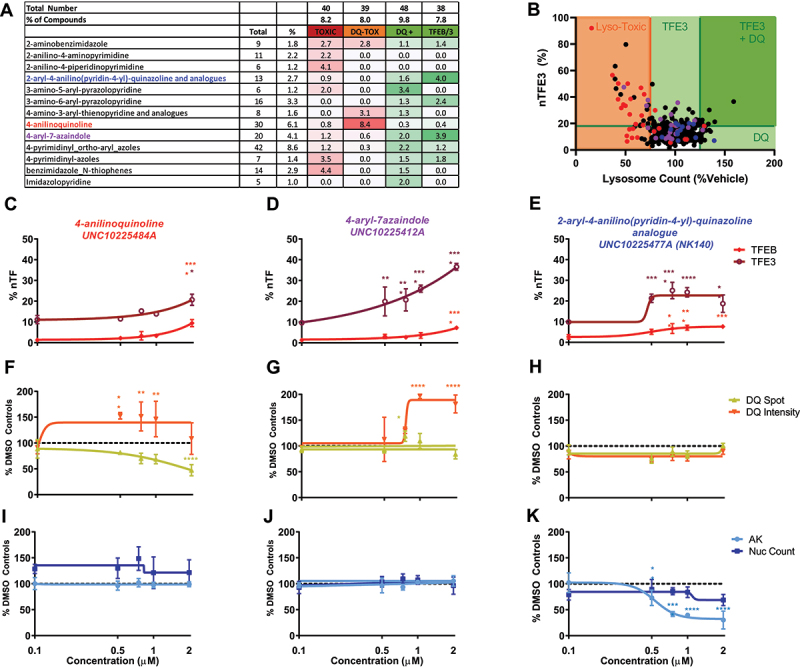
Patterns of activity enriched within identified chemotypes. (A) Chemotypes showing enrichment >2 against a particular biological output. The “2-aryl-4-anilino(pyridine-4-yl)-quinazoline and analogs” chemotype (blue) is enriched for TFEB and TFE3 translocation hits, “4-anilinoquinoline” chemotype (red) is enriched for lysosomal toxicity and the “4-aryl-azaindole” chemotype (purple) is enriched for both TFEB and TFE3 translocation and increased lysosome activity. (B) Correlation between %nTFE3 translocation and lysosome function is often associated with reduced lysosome function indicated by reduced lysosome count, particularly noticeable in the “4-anilinoquinoline” chemotype (red). (C-E) % nTF indicates the nuclear TFEB and TFE3 translocation, (F-H) DQ Red BSA degradation and (I-K) AK and nuclear count for toxicity were measured after SH-SY5Y cells were exposed to increasing concentrations of compound for 24 h (n = 1 due to compound availability; error bars = s.d.). (**C, F, I**) The compound UNC10225484A of the “4-anilinoquinoline chemotype” shows a modest TFEB and 3 translocation at 2 µM but a correlated drop in DQ Red BSA spot count with no indication of cellular toxicity. (**D, G, J**) The compound UNC10225412A of the “4-aryl-azaindole” chemotype shows strong TFE3 translocation and an increase in DQ Red BSA intensity with no toxicity. (**E, H, K**) The compound UNC10225477A of the “2-aryl-4-anilino(pyridine-4-yl)-quinazoline and analogs” chemotype shows modest TFEB and TFE3 translocation with no increases in DQ Red BSA intensity or cellular toxicity. AK is reduced, suggesting either decreased cell death or off-target inhibition of AK enzyme. n = 1 for all above assays, mean ±s.d. *p < 0.05, **p < 0.01, *** p < 0.001, **** p < 0.0001 Two-way ANOVA with Dunnett’s multiple comparisons test.

The 490 small molecule inhibitors screened within PKIS2 represent 54 diverse chemotypes. By determining the percentage of the library represented by each chemotype and the number of compounds falling into each hit category it is possible to identify whether any chemotypes have enriched activity across the assays. Chemotypes with >2-fold enrichment across any category are shown in Figure 3A (full data available in Table S2). The highest chemotype enrichment (8.4 fold) was that of 4-anilinoquinolines for lysosomal toxicity, with 20/30 anilinoquinoline compounds showing inhibition of the DQ Red BSA assay. There was a strong correlation between loss of lysosome function, indicated by DQ Red BSA signal and TFE3 translocation, particularly within the *4-anilinoquinoline* chemotype which has structural similarity with chloroquine (Figure 3B). The number of DQ Red BSA puncta per cell was reduced by increasing concentrations of these compounds, exemplified by UNC10225484A, which showed some TFEB and TFE3 translocation (Figure 3C) with reduction in active lysosome spots (Figure 3F) and no cellular toxicity (Figure 3I).

The *4-aryl-7-azaindole* chemotype was enriched for both DQ Red BSA activity (2 fold) and TFEB and TFE3 translocation (3.9 fold). The active compounds within this chemotype are AKT inhibitors with nanomolar potency [20] but also show high levels of promiscuity across the KINOME*scan* data [19]. Structurally diverse compounds within this chemotype that were developed as IKK and ROCK1 inhibitors, as previously reported [21], were inactive in our assays. Increasing concentrations of active 4-aryl-7-azaindole compounds, as exemplified by UNC10225412A, induced both an increase in DQ Red BSA fluorescence intensity, with a small reduction in puncta count and translocation of TFEB and TFE3 without cellular toxicity (Figure 3D,G,J).

Another chemotype enriched for TFEB and TFE3 translocation was 2*-aryl-4-anilino(pyridin-4-yl)-quinazoline and analogs* (4 fold). A subgroup of this chemotype induced a modest translocation of TEFB and TFE3 at 0.5 µM, which plateaued and remained stable up to 2 µM (Figure 3E) and no reduction in the DQ Red BSA assay outputs (Figure 3H). While this compound showed no decrease in nuclear count, it shows a reduction of the AK signal indicating a potential interaction with the assay (as toxicity would be indicated by an increase in signal) (Figure 3K).

### TFEB and TFE3 activation is preserved across an expanded analog series

**Figure 4.**
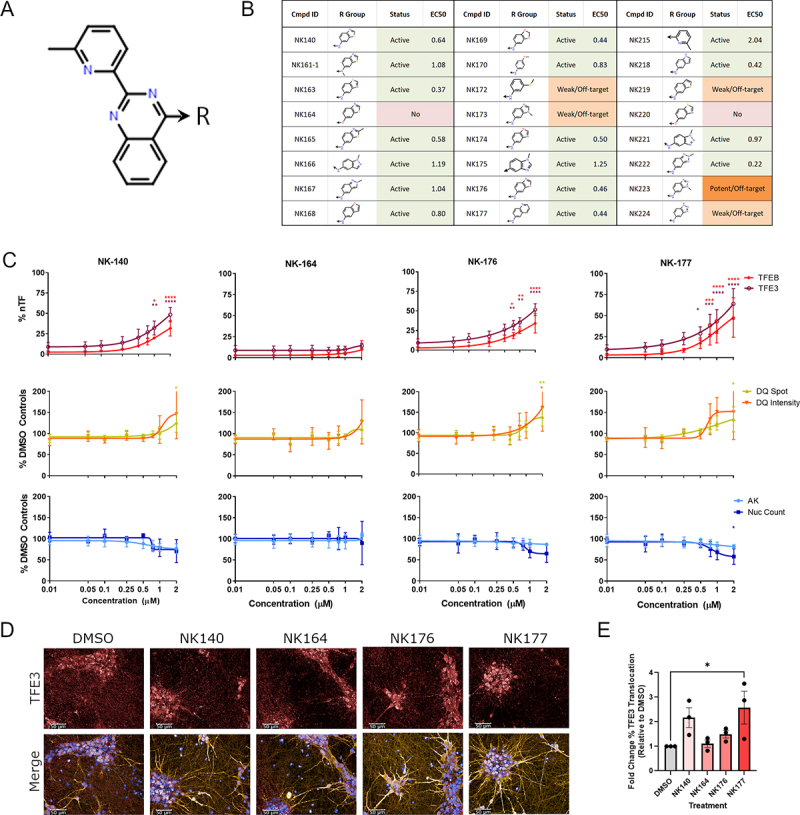
A novel chemical series which modulates TFEB and TFE3 regulation and lysosome activity. (A, B) a series of novel analogs of NK140 was assessed in concentration response at 24 h treatment time for toxicity and activity across the panel of assays. (C) TFEB and TFE3 translocation was confirmed to be induced by the hit compound NK140 but not the negative compound NK164. TFEB and TFE3 translocation is also induced by the analogs NK176 and NK177. DQ Red BSA signal was seen to be slightly, but not-significantly, increased. NK140, NK176 and NK177 show a small reduction in nuclear count from 0.75 µM without increases in AK, suggesting a small anti-proliferative effect over 48 h treatment. n = 3 for all above assays, mean ± s.d. *p < 0.05, **p < 0.01, *** p < 0.001, **** p < 0.0001 Two-way ANOVA with Dunnett’s multiple comparisons test. (D) iPSC-derived i3Neurons treated with compounds as shown were stained with DAPI (blue), and immunostained for MAP2 (yellow) and TFE3 (red). Scale bars: 50 µm. (E) Nuclear translocation of TFE3 was quantified, showing a significant relocalization by the active compound NK177 (n = 3 for all above assays, mean ± s.d.; One-way ANOVA with Dunnett’s multiple comparisons test).

In addition to the original confirmed hit compound (UNC10225477A also referred to as NK140) and three other *2-aryl-4-anilino(pyridin-4-yl)-quinazoline* compounds from the PKIS2 set (NK166; NK215; NK224), a SAR-analysis was performed on further 20 structurally diverse analogs which were synthesized and tested for induction of TFEB and TFE3 translocation after 24 h of treatment (Figure 4A, B; Figure S5A; Table S3). The activity of a new synthesis of the original hit NK140 was reconfirmed with an estimated cellular EC_50_ against TFE3 of 0.65 µM. Of particular note is the analog NK164, which differs from the original compound by only an oxygen substitution which should ablate kinase inhibition activity, and does not translocate TFEB or TFE3. Of the remaining analogs, 17 had concentration response curves similar to that of NK140 with variable EC_50_ values. Several analogs, including NK176, showed similar or increased potency to NK140 (EC_50_ = 0.46 µM) with no detectable cellular or lysosomal toxicity at 24 h of treatment. In addition to analogs which closely replicate the activity of the original hit compound which plateaued at ~1 µM, some compounds (e.g. NK177) demonstrated increasing levels of TFEB and TFE3 translocation without plateauing, representing increased potency or activity against a different target. Other analogs showed either markedly weaker effect across the TFEB and TFE3 assays, or some off-target activity only present at higher concentrations (~5 µM) often observed with associated toxicity.

The effect of treatment with these selected exemplar compounds, NK140, NK164, NK176 and NK177, at 48 h of treatment demonstrated elevated TFEB and TFE3 translocation in positive compounds (excluding NK164) which did not plateau beyond 1 µM and an additional increase in DQ Red BSA intensity not seen previously after 24 h of treatment (Figure 4C). Furthermore, increased translocation of TFEB to the nucleus upon treatment with NK140 and NK176, but not the inactive NK164, was confirmed in a cellular fractionation assay (Figure S5B and C).

We next tested our original compound (NK140), the inactive analog (NK164), and the improved derivatives (NK176, NK177) in i^3^Neurons – a rapidly inducible, functional glutamatergic cortical neuron system [22,23] – following an optimized two-step differentiation protocol [24]. Although the data with i^3^Neurons are more variable than the highly uniform SH-SY5Y cultures, we confirmed a trend toward upregulation of TFE3 translocation on treatment with the original active compound NK140, which reached statistical significance (*p < 0.05) with the derivative analog NK177 (Figure 4D and 4E).

#### The activity of quinazoline analogs is independent of *PRKD.*

The enriched chemotype *2-aryl-4-anilino(pyridin-4-yl)-quinazoline analogs* from the PKIS2 library comprises compounds originally derived from quinazoline as part of a series developed to identify potent and selective ALK5/TGFBR1 inhibitors [25]. However, the compounds that were the strongest hits for TFEB and TFE3 activation (GW867587 and GW873004) showed the lowest TGFBR1 binding (0.59 µM and 0.65 µM, respectively). Conversely, compounds within this chemotype which did not show TFEB and TFE3 activation, GSK204919 and GSK257997, showed higher affinity for TGFBR1 binding (0.02 µM and 0.025 µM respectively). This, and TGFBR1 binding data for the novel analogs in this study, which confirmed no correlation, suggests that TGFBR1 is unlikely to be the relevant kinase for induction of TFEB (See Table S3). In contrast, KINOME*scan* data for compounds within the chemotype revealed that PRKD1 and PRKD2 (protein kinase D; PRKD) are the only kinases with binding >90% (Table S4). KINOMEscan data are available for a select number of the novel analogs; however, none of the tested kinases were inhibited to a high degree across the active compounds, including PRKD1 and PRKD2, making target deconvolution challenging (Table S5).

**Figure 5.**
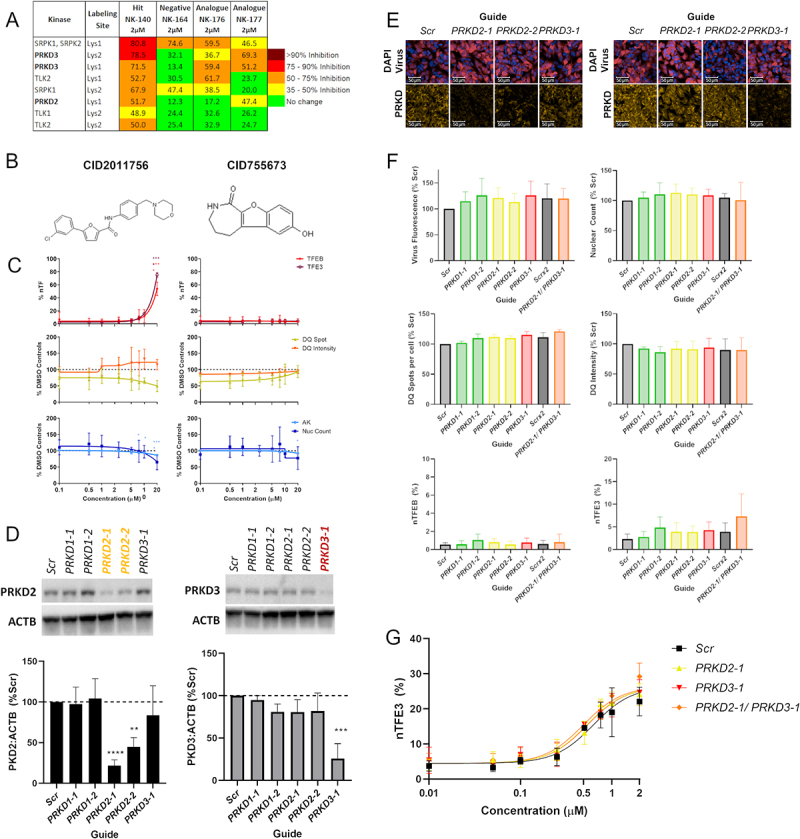
Quinazoline analog series activity is independent of PRKD. (A) The top results from the KiNativ in-cell kinase binding activity, highlight specific binding to PRKD2 and PRKD3. (B) Structures of commercially available protein kinase D inhibitors CID2011756 and CID755673. (C) %nTF indicates the percentage of nuclear TFEB and TFE3 translocation, DQ Red BSA degradation and AK and nuclear count for toxicity were measured in SH-SY5Y cells after exposure to increasing concentrations of CID2011756 and CID755673 compounds for 24 h. CID2011756 shows some TFEB and TFE3 translocation only at 20 µM where there is also some reduction of DQ Red BSA signal and toxicity. CID755673 shows no TFEB and TFE3 translocation, alteration in DQ Red BSA signal or toxicity. (D) Using CRISPRi, PRKD2 and PRKD3 were knocked down to ~21 and 25% of basal levels, respectively, as measured by western blotting. (E) sgRNA guides were co-expressed with iRFP670, allowing visualization of vector transduction alongside PRKD2 and PRKD3 knockdown using immunocytochemistry. Scale bars: 50 µm. (F) Guide fluorescence, nuclear count, DQ Red BSA active spot count and correct fluorescent intensity and TFEB and TFE3 translocation showed no differences between targeting guides and appropriate Scr controls. (G) Cells treated with Scr or *PRKD2* or *PRKD3* targeting guides were exposed to increasing concentrations of NK140 compound; however, demonstrated no increased activity in the TFE3 translocation assay (n = 3 biological replicates, error bars = s.d). n = 3 for all above assays, mean ± s.d. *p < 0.05, **p < 0.01, *** p < 0.001, **** p < 0.0001 One-way ANOVA with Dunnett’s multiple comparisons test.

As KINOME*scan* is an *in vitro* assay, and as different cell types have variable kinase expression profiles we sought to determine the kinase binding profile in our SH-SY5Y TFEB-relocalization cell model. Cells were treated with compounds of interest for 2 h and cell lysates were then incubated with KiNativ™ kinase probes, which are prevented from binding to a cellular kinase if that candidate kinase is already bound by a compound [26]. Bound kinases were pulled down and analyzed by mass spectrometry to determine the *in situ* kinase binding profile for each compound. These results confirm that PRKD2 and PRKD3 are bound by the TFEB-activating compounds NK140, NK176 and NK177 but, importantly, not by our negative compound NK164 (Figure 5A; Full Data Table S6). In contrast, other kinases such as SRPK1/2 and TLK1/2 are bound by either the inactive compound (NK164) or are not shown to be bound by all active compounds. Together, these data suggest that the chemical series elicits its TFEB and TFE3 relocalization effects via PRKD2 and PRKD3.

PRKD is a family of serine/threonine kinases of the Ca^2+^/Calmodulin-dependent kinase superfamily. To investigate whether PRKD is the relevant target of the quinazoline analogs we first tested additional commercially available cell-active PRKD inhibitors (CID2011756, CID755673, CRT0066101; Tocris Bioscience) (Figure 5B and Figure S6A). The compounds are structurally diverse, and are reported to have nanomolar potency for PRKD and high selectivity against other kinase targets. Interestingly, these compounds gave discrepant results across our assays. CRT0066101 (IC_50_ values = 1, 2 and 2.5 nM for PRKD1, PRKD3 and PRKD2, respectively [27]) showed pronounced cellular toxicity, preventing accurate assessment of TFEB and TFE3 translocation independent of toxicity (Figure S6B). CID2011756 (IC_50_ values = 0.6, 0.7 and 3.2 μM for PRKD2, PRKD3 and PRKD1, respectively [28]) induced TFEB and TFE3 translocation only alongside marked lysosomal and cellular toxicity at 20 µM (Figure 5C). CID755673 (IC_50_ values = 0.182, 0.280 and 0.227 μM at PRKD1, PRKD2 and PRKD3 respectively [29]) showed no effect across these assays (Figure 5C).

Given the promiscuity of many kinase inhibitors, we therefore sought to clarify if PRKD regulates TFEB and TFE3 using the CRISPRi genetic knockdown approach against individual targets. We generated an SH-SY5Y cell line containing dCAS9-KRAB and delivered sgRNA guides co-expressed with an iRFP670 fluorescent marker and blasticidin resistance on a lentiviral backbone (Figure S6C). In agreement with the KiNativ™ data, we found PRKD1 protein to be undetectable in SH-SY5Y cell lysate despite being present in other cell-types (Figure S6D). We therefore used CRISPRi and sgRNA targeting *PRKD2* and *PRKD3* to reduce protein expression to 21 and 25%, respectively, of the basal levels in Scrambled-Guide (Scr) treated cells, with the *PRKD1* guides serving as additional controls showing no decrease in PRKD2 or PRKD3 protein (Figure 5D; Figure S6E). As the guides are co-expressed with iRFP670 far-red fluorescent protein, we could visualize vector transfection and assessment of PRKD2 and PRKD3 knockdown via immunocytochemistry (Figure 5E). Each sgRNA was transduced into cells at similar levels and the nuclear count after blasticidin selection was consistent across different guides. However, knockdown of PRKD2, PRKD3 or simultaneous PRKD2 and PRKD3 knockdown did not result in significant alterations in DQ Red BSA signal or TFEB or TFE3 activation (Figure 5F). To rule out that residual PRKD activity from incomplete knockdown was sufficient to prevent TFEB and TFE3 translocation, we sought to determine whether knockdown of PRKD2 or PRKD3 was able to increase TFE3 translocation in response to the PRKD binding compounds. SH-SY5Y cells were treated with increasing concentrations of NK140 compound after treatment with either Scr, *PRKD2* or *PRKD3* targeting guides. Crucially, TFE3 nuclear translocation in response to NK140 was not altered by knockdown of PRKD (Figure 5G), confirming that PRKD inhibition is not related to the induction of TFEB or TFE3 by the quinazoline chemical series.

### Quinazoline analogs induce CLEAR gene expression and reduce HTT protein

**Figure 6.**
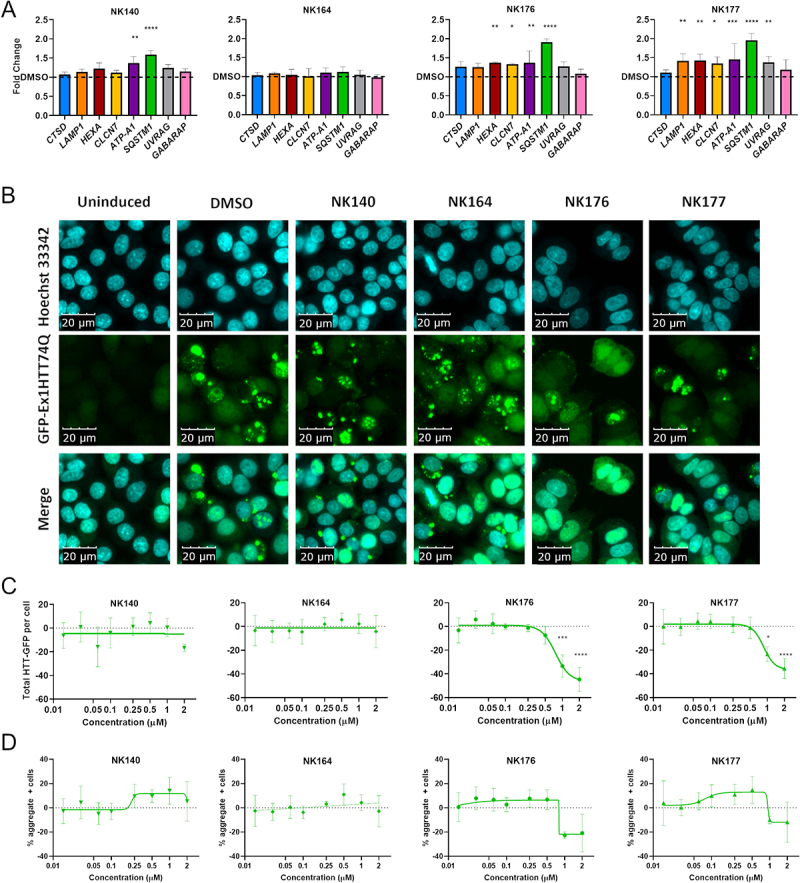
Upregulation of CLEAR genes and clearance of HTT aggregates. (A) Expression of eight CLEAR genes, *CSTD, LAMP1, HEXA, CLCN7, ATP6V0A1* (ATP-A1), *SQSTM1, UVRAG* and *GABARAP* was assessed after treatment with 1 µM compound for 48 h and showed significant increase of multiple genes with NK176 and NK177 treatment. (B) GFP-Ex1HTT74Q HTT expression can be induced, and visualized as bright puncta where aggregates are formed. Nuclei stained with Hoechst 33,342. (C) Total-GFP fluorescence shows reduction in signal after treatment with either NK176 or NK177 at 1 and 2 µM (n = 3). (D) The percentage of cells bearing aggregates is reduced at higher concentrations, but the difference is non-significant due to the variability in the assay output. n = 3 except for NK176 and NK177 1 and 2 µM where n = 2. * p < 0.05, *** p < 0.0, **** p < 0.0001 Two-Way ANOVA with Dunnett’s multiple comparisons test.

We then tested the downstream phenotypic effect of the analog series in a target-agnostic manner to investigate if the TFEB and TFE3 relocalization we observed resulted in downstream engagement of the CLEAR gene network and potentiation of protein clearance. We quantified the expression of a panel of lysosomal and autophagy CLEAR genes (*CTSD, LAMP1, HEXA, CLCN1, ATP6V0A1, SQSTM1, UVRAG* and *GABARAP*) after 48 h of treatment with DMSO, NK140, NK164, NK176 and NK177 by RT-qPCR. We confirmed an upregulation of a number of CLEAR genes after treatment with NK140 and to a greater extent with active analogs NK176 and NK177 (Figure 6A), but not the inactive compound (NK164). We assessed autophagic flux by quantifying LC3-II levels using western blotting in compound-treated cells in the presence or absence of the lysosomal inhibitor bafilomycin A_1_. Although bafilomycin A_1_ had a significant effect (p < 0.01, two-way ANOVA) on LC3-II levels as expected, neither active nor inactive analogs significantly affected autophagic flux (P > 0.05, two-way ANOVA), with the exception of NK177 which demonstrated a mild impairment of autophagic flux (Figure S6F). Given the lack of correlation between the ability of the compounds to upregulate CLEAR genes, and changes in LC3-II levels, the action of the compounds was therefore independent of autophagic flux.

We next tested the ability of the original hit NK140 and analogs NK176 and NK177 to degrade disease-relevant protein aggregates using a cellular model of HTT aggregation. The presence of HTT aggregates upon induction of expression of a GFP-tagged *HTT* exon 1 containing a 74-repeat polyglutamine-expansion (GFP-Ex1HTT74Q) was visualized by imaging fluorescent puncta (Figure 6B). Treatment with either the original hit compound NK140, or the negative control NK164 caused no reduction in fluorescent aggregates. However, treatment with the more potent TFEB and TFE3 inducers NK176 and NK177 showed a concentration-dependent clearance of total GFP-HTT (Figure 6C and 6A) reduction, although not reaching significance, of the number of cells bearing aggregates at the highest concentrations (Figure 6D). These data confirm the utility of this TFEB and TFE3-inducing chemical series in proteinopathies.

## Discussion

Lysosome dysfunction and autophagic impairment have been linked to the pathogenesis of many neurodegenerative diseases, such as Parkinson disease (PD), Huntington disease (HD) and Alzheimer disease (AD) [30]. This dysfunction manifests through impaired autophagic signaling, elevated number of autolysosomes with undegraded cargo, compromised lysosomes and accumulation of damaged organelles such as mitochondria. Without the successful degradation of aggregation-prone proteins and dysfunctional organelles, neurons are left vulnerable to cellular stress, mitochondrial-derived oxidative stress and eventual death.

TFEB function is perturbed across many neurodegenerative diseases and TFEB overexpression studies have suggested it to be a promising therapeutic target to prevent neuronal loss. In PD, AAV-mediated overexpression of TFEB prevented degeneration of dopaminergic neurons in a rat model of α-synuclein induced toxicity [31]. Overexpression of TFEB in both MAPT/Tau and APP-PSEN1/PS1 mice models shows beneficial effects in reducing Tau pathology, reducing APP and Aβ production, ultimately resulting in reduced pathology and rescuing behavioral phenotypes [32,33]. Overexpression of PPARGC1A/PGC-1α and activation of TFEB expression, or direct TFEB overexpression and subsequent upregulation of the CLEAR gene network eliminated HTT protein aggregates and reduced neurotoxicity in HD transgenic mice and cell models [34].

While overexpression of TFEB has proved successful in ameliorating disease pathology in many neurodegenerative conditions, few small molecule activators of TFEB are known [35]. Trehalose has several suggested mechanisms of action, which include activation of TFEB via AKT inhibition [14]. Trehalose has been extensively studied in many cellular and *in vivo* models of neurodegeneration and shows effective induction of autophagy and reduction of disease pathology [36]. However, in some cell models trehalose blocks autophagic flux from autophagosome to autolysosome, leaving some unanswered questions as to whether trehalose will make an appropriate therapy for neurodegenerative diseases [37].

We have established a robust high-content screening platform to identify novel compounds and targets that induce translocation of endogenous TFEB and TFE3 to the nucleus and promote lysosome function in SH-SY5Y cells without the reliance on overexpression vectors. As TFEB and TFE3 expression are variable across different cell types and may have different regulatory roles in different tissues [38,39], measuring translocation of both endogenous TFEB and TFE3 ensures that compounds regulating both transcription factors are selected for further development.

The high level of cellular and lysosomal toxicity induced by some compounds and correlated to TFEB and TFE3 translocation has demonstrated the necessity to account for these effects during the primary screening stage. Of 490 compounds, 79 (~16%) induced either cellular toxicity or lysosomal inhibition, often with induced TFEB and TFE3 translocation. As an exemplar, the 4-anilinoquinoline compounds from the PKIS2 library which were toxic to lysosomes were thus excluded from further analysis. As the negative impact of lysosomotropic compounds increases at higher concentrations, it was necessary to identify and exclude these compounds in compound screens at higher concentrations, prior to extensive study in resource-heavy concentration-response and secondary assays. It is also evident that the strongest TFEB and TFE3 translocation is most often concomitant with severe lysosomal inhibition, as evident with chloroquine, thereby making any strategy relying on compounds selection of the strongest translocation more likely to increase the proportion of compound hits with lysosome inhibition if this is not taken into consideration.

The in-depth characterization of the PKIS2 library compounds presented a valuable opportunity to validate our newly established screen. Using a multiparameter screen of TFEB and TFE3 translocation, lysosome function and cellular toxicity, we have identified a set of AKT inhibitors (*4-aryl-7-azaindole* chemotype) which strongly activate TFEB and TFE3 and enhance lysosomal function. While enrichment of this chemotype against a target known to regulate TFEB provides good proof of principle that the assay is able to identify compounds which regulate TFEB, the *4-aryl-7-azaindole* compound series are known to inhibit a large number of other kinases on the KINOME*scan* data and thus do not represent a good opportunity to develop selective inhibitors.

We confirmed that *2-aryl-4-anilino(pyridin-4-yl)-quinazoline analogs* compounds induce TFEB and TFE3 translocation. Active compounds within this series were highly selective according to *KINOMEscan* data, showing low binding activity against >95% of screened kinases and high affinity only for PRKD1 and PRKD2 and binding to only a small number of kinases on the KiNativ™ cellular kinase binding assay. We then developed advanced CRISPRi methodology for evaluating potential compound targets. Although induction of TFEB and TFE3 translocation by the quinazoline analogs appears to be independent of PRKD inhibition, there are several potential reasons why CRISPRi knockdown may differ from the effects of pharmacological inhibition. First, treatment with a compound may only inhibit the catalytic subunit of a target protein, whereas knockdown with CRISPRi will prevent protein expression and remove any structural role a protein may have. Second, it is challenging to match the time taken for a pharmacological inhibitor to act, which can be very fast, with the timing of knockdown treatments which may take days to reduce mRNA and protein levels and will depend on mRNA and protein stability. Third, an incomplete knockdown may leave residual activity, preventing the presentation of any phenotypes associated with complete loss or inhibition of the target. Finally, there may be additional effects from unintended off-target silencing of proteins that differ from pharmacological off-target effects.

Finally, we note that RT-qPCR proved to be a more sensitive, quantitative and accurate method for detecting upregulation of CLEAR gene expression after TFEB and TFE3 relocalization as compared to semi-quantitative protein detection in whole cell lysates by western blots. Discrepancies between mRNA and protein levels are not unusual and may be because the turnover of proteins is likely on substantially different timescale than of gene transcripts. In addition, both post-transcriptional regulation and post-translational regulation (e.g. protein folding and glycosylation, particularly of lysosomal membrane proteins such as LAMP1) may affect protein stability.

Overall, we have demonstrated that it is possible to identify novel chemical series which induce TFEB and TFE3 translocation, upregulate genes within the CLEAR network and promote clearance of toxic proteins. Kinases represent tractable targets for drug discovery and this work highlights the presence of additional kinase targets to discover in the lysosomal biogenesis pathways and encourages us to attempt to identify the target of the quinazoline series.

## Materials and methods

### Cell culture

SH-SY5Y cells were cultured at 37°C with 5% CO_2_ in Dulbecco’s modified Eagle’s Medium (DMEM)-F12 (ThermoFisher Scientific/Gibco, 21,331–020) supplemented with 10% fetal bovine serum (FBS; ThermoFisher Scientific/Gibco, 10,270,106), 2 mM L-glutamine (ThermoFisher Scientific/Gibco, A2916801) and 100 U ml^−1^ penicillin and 100 mg ml^−1^ streptomycin (ThermoFisher Scientific/Gibco, 15,140,122). For screening, cells were plated at 4000 cells per well in 384-well Cell Carrier Ultra (Perkin Elmer, 6,057,302) imaging plates coated with 0.1% gelatine (Sigma Aldrich, G9391) and incubated overnight prior to compound treatment.

hiPSCs were maintained under feeder-free conditions in a 37°C, 5% CO_2_ tissue culture incubator on tissue culture treated dishes coated with growth factor-reduced Matrigel (Corning, 354,277) and fed every 1–2 days with Essential 8 medium (Life Technologies, A1517001), as needed. Accutase (STEMCELL Technologies, 07920) was used to enzymatically dissociate hiPSCs into single cells, and 0.5 mM EDTA was used for routine dissociation to maintain colony growth. To promote cell survival during passaging, cells were passaged with the ROCK/p160-Rho-associated coiled coil kinase inhibitor Y-27632 (10 mM; Selleckchem, S1049). hiPSC-derived i^3^Neurons were induced and differentiated according to Fernandopulle et al. [24] and replated on high-content 96-well dishes (PerkinElmer, CellCarrierUltra-96) by day 3. Media changes were performed every other day with Cortical Neuron Culture Medium (CM) [24] until day 10, when translocation assays were performed.

### Compound treatment

The PKIS2 Library was supplied by The Structural Genomics Consortium at the University of North Carolina at Chapel Hill. The PKIS2 library was prepared at 1 mM concentration in DMSO in 384-Well Low Dead Volume (LDV) source plates and were dispensed using the ECHO 550 (Labcyte; San Jose, CA, USA) into a total well volume of 40 µL. The primary screen was performed at 1 µM for 24 h, in duplicate for lysosomal assays (on duplicate plates) and as single replicates for immunocytochemistry for TFEB or TFE3. Each concentration on a dose response was performed in triplicate between 0.01 and 2 µM for 24 h. Additional analogs and commercially available compounds were assayed up to 5 and 20 µM respectively. The control compounds selected for use with the assays were the class I HDAC inhibitor CI-994 (Tacedinaline; Tocris Bioscience, 2952) at 10 µM and the lysosomal inhibitor chloroquine at 20 µM (Sigma-Aldrich, C6628).

### AK (adenylate kinase) assay

After incubation with compounds for 24 h, 10 µL of media was sampled and transferred to an independent white-walled 384-well plate (Greiner Bio-One, 781,080). Toxicity was assessed using the ToxiLight^TM^ Nondestructive Cytotoxicity BioAssay Kit (Lonza, LT17-217) using the Pherastar (BMG Labtech, Ortenberg, Germany) for luminescence measurement. Total cell lysis with 5 µL 5% Triton-X 100 (Sigma-Aldrich, T8787) is used as a positive control for the assay and toxicity is expressed as a percentage of luminescence under DMSO conditions.

### Lysosomal functional assay

Lysosome function was assessed using DQ™ Red BSA reagent (ThermoFisher Scientific/Invitrogen, D12051). After removal of media for the toxicity measurement, each well was supplemented with 1.25 µL of NucBlue™ Live ReadyProbes™ (ThermoFisher Scientific/Invitrogen, R37605) in solution with DQ Red BSA to a final concentration of 35 µg/mL. Cells were incubated at 37°C for 2 h without media change (compounds still present). Prior to imaging, cells were washed with Dulbecco’s Modified Phosphate Buffer (DPBS; ThermoFisher Scientific/Gibco, 14,040,117) and fresh media replaced. Cells were imaged using the OperaPhenix (PerkinElmer, Waltham, MA, USA) using the blue channel (375-nm laser and 435- to 480-nm filter) for NucBlue and orange/red channel (561-nm laser and 570- to 630-nm filter) for DQ Red BSA. The Harmony image analysis software (Perkin Elmer) was used to identify nuclei and DQ Red BSA Red positive spots. Nuclei count was taken as a measure of cellular growth and toxicity. The number and intensity of DQ Red BSA spots was considered as a measure of active lysosome function (see Figure S4 for analysis protocol).

### TFEB and TFE3 immunocytochemistry

After the required compound incubation, media was removed from the wells and the cells were fixed in 4% paraformaldehyde (PFA; ThermoFisher Scientific/Pierce, 28,908) for 20 min. Cells were washed in DPBS 3 times and blocked and permeabilized in a solution of 5% normal donkey serum (NDS; Sigma-Aldrich, 566,460), 2% bovine serum albumin (BSA; Sigma-Aldrich, A7030) and 0.5% Triton X-100 in DPBS for 1 h. The primary TFEB antibody (Cell Signaling Technology, 4240) was incubated at 1:1000 dilution for 16 h overnight at 4°C. The primary TFE3 antibody (Atlas Antibodies, HPA023881 Lot Q115942) was incubated at 1:1000 dilution for 2 h at room temperature. TFEB and TFE3 staining was performed on parallel plates because both primary antibodies are raised in rabbit. Cells were washed twice with DPBS and incubated with 1:1000 diluted donkey anti-rabbit 647 Alexa Fluor secondary antibody (ThermoFisher Scientific/Invitrogen, A-31573) for 2 h at room temperature with NucBlue™ Live ReadyProbes™ (1 drop/mL). Cells were washed 3 times and fresh DPBS added. Cells were imaged using the OperaPhenix (PerkinElmer) using the blue channel (375-nm laser and 435- to 480-nm filter) for NucBlue and far-red channel (640-nm laser and 650- to 760-nm filter) for TFEB or TFE3. The Harmony image analysis software (Perkin Elmer) was used to identify nuclei TFEB or TFE3 positive nuclei, expressed as a percentage of total cell number (See Figure S2 and S3 for analysis protocols). Mean values and standard deviation of DMSO control wells on the plate were calculated. Compounds with translocation > Control Mean +3 s.d were deemed hit compounds.

### RNA extraction and RT-qPCR analysis of CLEAR gene expression

SH-SY5Y cells were treated with compounds in 12-well or 24-well plates (Corning Costar, CLS3513-50EA). Cell pellets were harvested and RNA was extracted using the RNeasy Mini Kit (QIAGEN, 74,104) according to manufacturer’s instructions. cDNA was generated from 500 ng RNA using Superscript III Reverse Transcriptase (Thermofisher Scientific, 18,080,085). Expression of the CLEAR genes *CTSD, HEXA, LAMP1, CLCN7, ATP6V0A1, SQSTM1, UVRAG and GABARAP* were assessed using Fast SYBR Green Master Mix standard protocol (Thermofisher Scientific, 4,309,155), on a StepOnePlus™ Real-Time PCR System (Applied biosystems, Waltham, MA, USA). Primer sequences are shown in Table S7A and were designed in Primer3 or by OriGene Technologies and validated in house for efficiency and specificity.

### SDS-PAGE and western blotting

Cells were washed with cold PBS (ThermoFisher Scientific/Gibco, 10,010–015) and lysed in RIPA buffer (0.5% Triton X-100, 10 mM Tris/HCl, pH 8.0, 1 mM EDTA (Sigma Aldrich, E6511), 0.5 mM EGTA (Sigma Aldrich, E3889), 0.1% sodium dodecyl sulfate [SDS], 0.02% sodium deoxycholate (Sigma Aldrich, D6750), and 140 mM NaCl (Sigma Aldrich, S9625) supplemented with protease-inhibitor cocktail (Sigma Aldrich/Roche, 4,693,132,001) and phosphatase inhibitors (Roche/Sigma Aldrich, 4,906,845,001) for 30 min at 4°C. The lysates were centrifuged at 13,000 g for 15 min and the supernatants collected. Protein concentration was quantified using BCA reagent (Thermofisher Scientific, 23,252) and 4× SDS Laemmli buffer was added. The samples were boiled at 95°C for 5 min before SDS-polyacrylamide gel electrophoresis (SDS-PAGE) using Mini-PROTEAN® Precast Gels (Bio-Rad, 4,568,094). Ten micrograms of protein per lane were separated by SDS/PAGE and transferred to a PVDF membrane using the Trans-Blot Turbo Transfer System (Bio-Rad, Hercules, CA, USA). The following antibodies were used to visualize proteins using ChemiDoc Imaging System (Bio-Rad, Hercules, CA, USA): rabbit anti-PRKD1/PKCµ (PRKD1) (Cell Signaling Technology, 90,039) 1:1000; rabbit anti-PRKD2 (Cell Signaling Technology, 8188) 1:1000; rabbit anti-PRKD3 (Cell Signaling Technology, 5655) 1:1000 and HRP-conjugated anti-ACTB/β-actin (Abcam, ab49900) 1:50,000. For cellular fractionation experiments cells were collected and nuclear fractionation was carried out using NE-PER Nuclear and Cytoplasmic Extraction Reagents (ThermoScientific, 78,833), following the manufacturer’s protocol. Nuclear and cytoplasmic fractions were assessed with a LMNA/lamin A/C antibody (Santa Cruz Biotechnology, sc-7292) or GAPDH antibody (Abcam, ab9484), respectively.

### CRISPRi knockdown experiments

SH-SY5Y cells were transduced with pMH0006 (EF1a-dCas9-BFP-KRAB; Addgene, 135,448; deposited by Martin Kampmann & Jonathan Weissman) and subsequently flow sorted to obtain a BFP-positive cell population indicative of dCas9 expression. pMH0006 was a gift from Martin Kampmann & Jonathan Weissman (Addgene, 135,448; http://n2t.net/addgene:135448; RRID:Addgene_135,448) [40].

All sgRNA constructs were created by the ligation of annealed oligonucleotides into BstXI and BlpI sticky ends within plasmid J72, a modified pMK1334 (EF1a-Puro-T2A-2xmycNLS-WPRE-mU6-sgRNA). pMK1334 was a gift from Martin Kampmann (Addgene, 127,965; http://n2t.net/addgene:127965; RRID:Addgene_127,965). J72 was created by excising Puro-T2A-2xmycNLS via SphI and EcoRI from pMK1334 and insertion of iRFP670-T2A-BSD_Resistance via gBlock (IDT) (Figure S6C) [23]. sgRNA guide sequences can be found in Table S7B.

Lentiviruses were then produced for all sgRNA constructs by transfection into Lenti-X 293 T (Takara, 632,180) and subsequent supernatant collection, purification, and concentration (Lenti-X Concentrator; Takara, 631,231) for use in RT-qPCR, western blot, and live cell imaging.

### HTT aggregation assay

PC12 cells stably expressing GFP-tagged exon 1 of HTT with a 74-repeat polyglutamine-expansion (GFP-Ex1HTT74Q) under tetracycline-inducible promoter control [41]were maintained at 37°C and 5% CO_2_ in RPMI medium (Sigma Aldrich, R8758) supplemented with 10% horse serum (Sigma Aldrich, H1138), 5% fetal bovine serum, 1% Glutamine (Sigma Aldrich, G7513), 1% Pen-Strep (Sigma, P0781), 0.1 mg/mL G418 (ThermoFisher, 10,131,035), and 0.07 mg/mL hygromycin B (ThermoFisher, 10,687,010). Cells were plated at a density of 10,000 per well in 96-well black cycloolefin thin-bottom plates (Perkin Elmer, 6,055,300) coated with collagen IV (Sigma Aldrich, C5533). After 5 h, expression of GFP-Ex1HttQ74 was induced with doxycycline (100 ng/mL; Sigma Aldrich, D3447). After 16 h of induction, compounds (final concentrations 16 nM – 2 µM) were added in fresh medium with doxycycline (100 ng/ml). Final concentration of DMSO (Sigma Aldrich, D8418) was 0.1% in all wells. After 48 h of incubation in the presence of compound, cells were washed once in PBS and fixed with 4% paraformaldehyde (Thermofisher Scientific, 28,908) supplemented with 10 µg/mL Hoechst 33,342 (ThermoFisher Scientific, H3570) for 15 min. Cells were then washed in PBS twice and images were acquired on a GE InCell 6000 High Content Imager (405 nm and 488 nm channels, 10 fields/well, 20 × 0.75NA objective) (GE Healthcare, Chicago, IL, USA). Image feature extraction was carried out using CellProfiler software [42]. Data aggregation, normalization and distance score calculations were carried out using StratoMineR software.

### Statistical analysis

Statistical analysis was carried out on GraphPad Prism 8. Statistical tests used and the significance values found are discussed in the figure legends. Data are expressed as mean ± SD. For primary screening, p < 0.01 was considered statistically significant for nuclear translocation; for all other data p < 0.05 was considered statistically significant.

## Supplementary Material

Supplemental Material
