# Supplementary material for: Multiparameter phenotypic screening for endogenous TFEB and TFE3 translocation identifies novel chemical series modulating lysosome function
Source: Autophagy. 2022 Jul 25;19(2):692–705. doi: 10.1080/15548627.2022.2095834 (PMC9851200; doi:10.1080/15548627.2022.2095834)
Supplement: Supplemental Material [file KAUP_A_2095834_SM5251.zip › Supplementary Figures R7.docx]

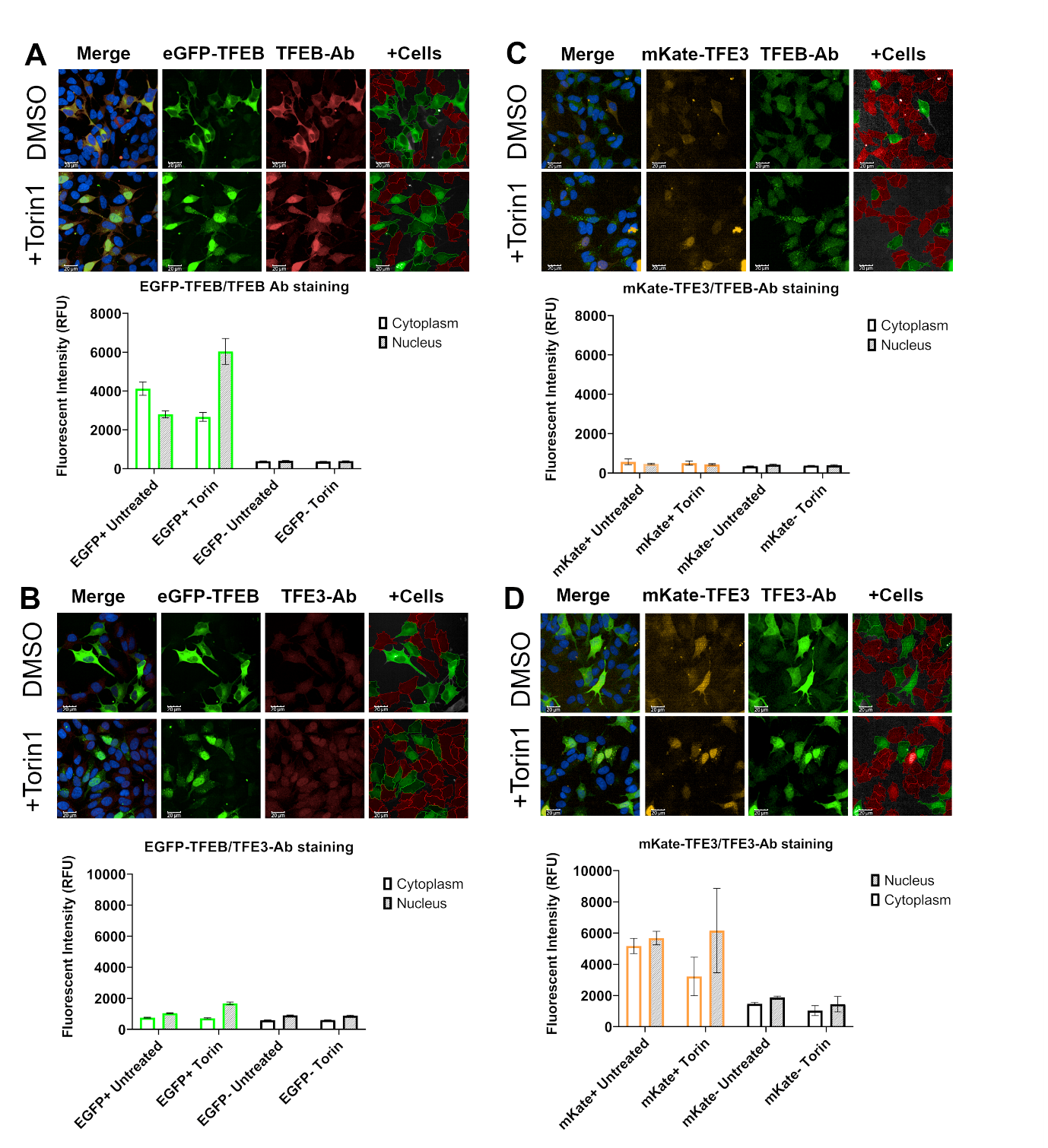


**Figure S1.** TFEB and TFE3 antibody validation with overexpressed proteins. SH-SY5Y were transfected with plasmids expressing either eGFP-TFEB or mKate-TFE3 and co-stained with either TFEB or TFE3 antibody. Nuclei are stained in the blue channel. Cells expressing EGFP-TFEB have increased staining using (**A**) the TFEB antibody, (**B**) but not with the TFE3 antibody. SH-SY5Y cells expressing mKate-TFE3 have (**C**) no increased staining with the TFEB antibody, but (**D**) greatly increased staining with the TFE3 antibody. Scale bars: 20 µm.


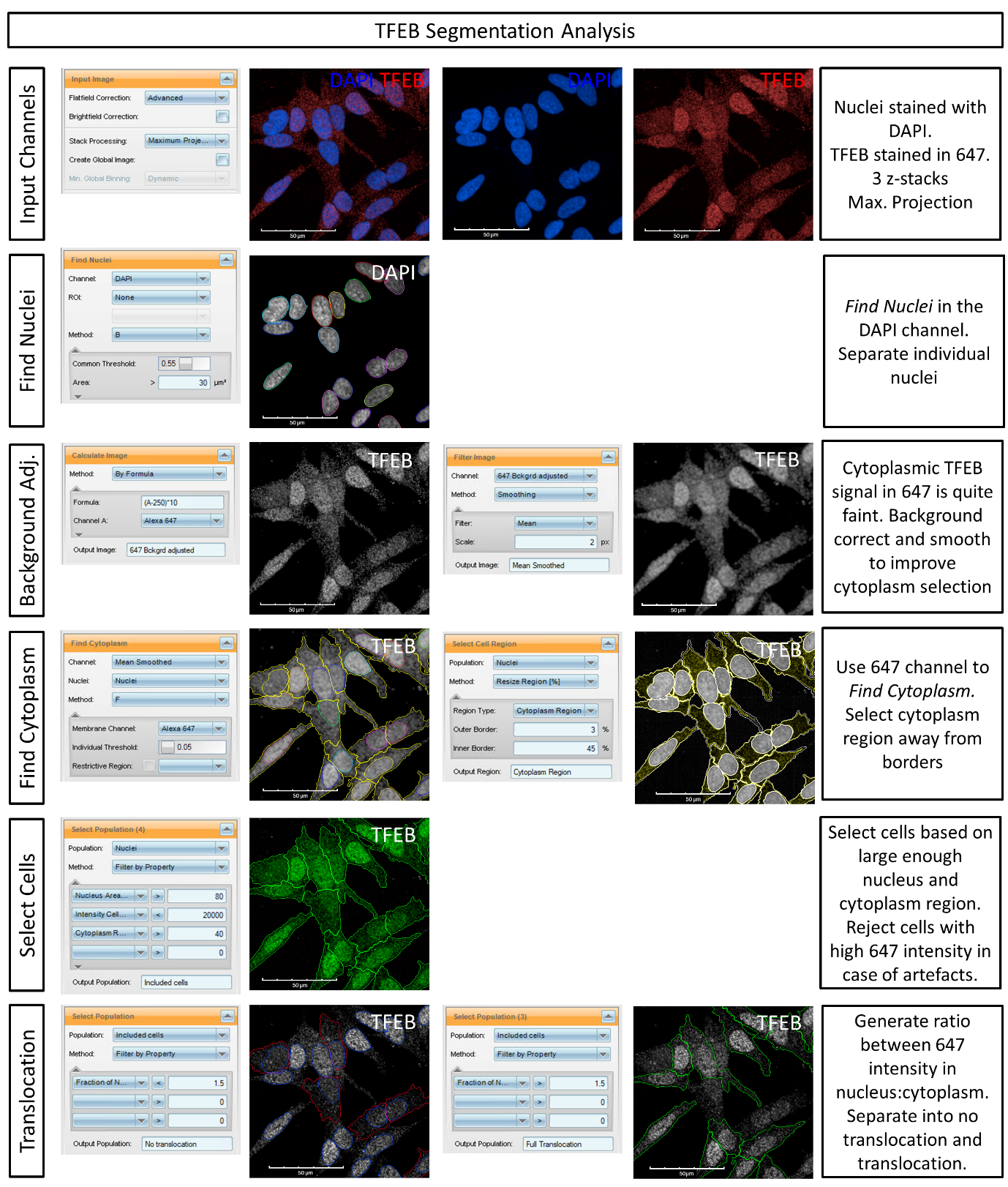


**Figure S2.** TFEB translocation segmentation analysis. Input images are processed through various image analysis building blocks on Harmony Image Analysis Software. Nuclei and Cytoplasmic Regions are identified. A ratio of the intensity of 647 in nucleus:cytoplasm is calculated and used to distinguish between cells with or without translocation of TFEB. Scale bars: 50 µm.


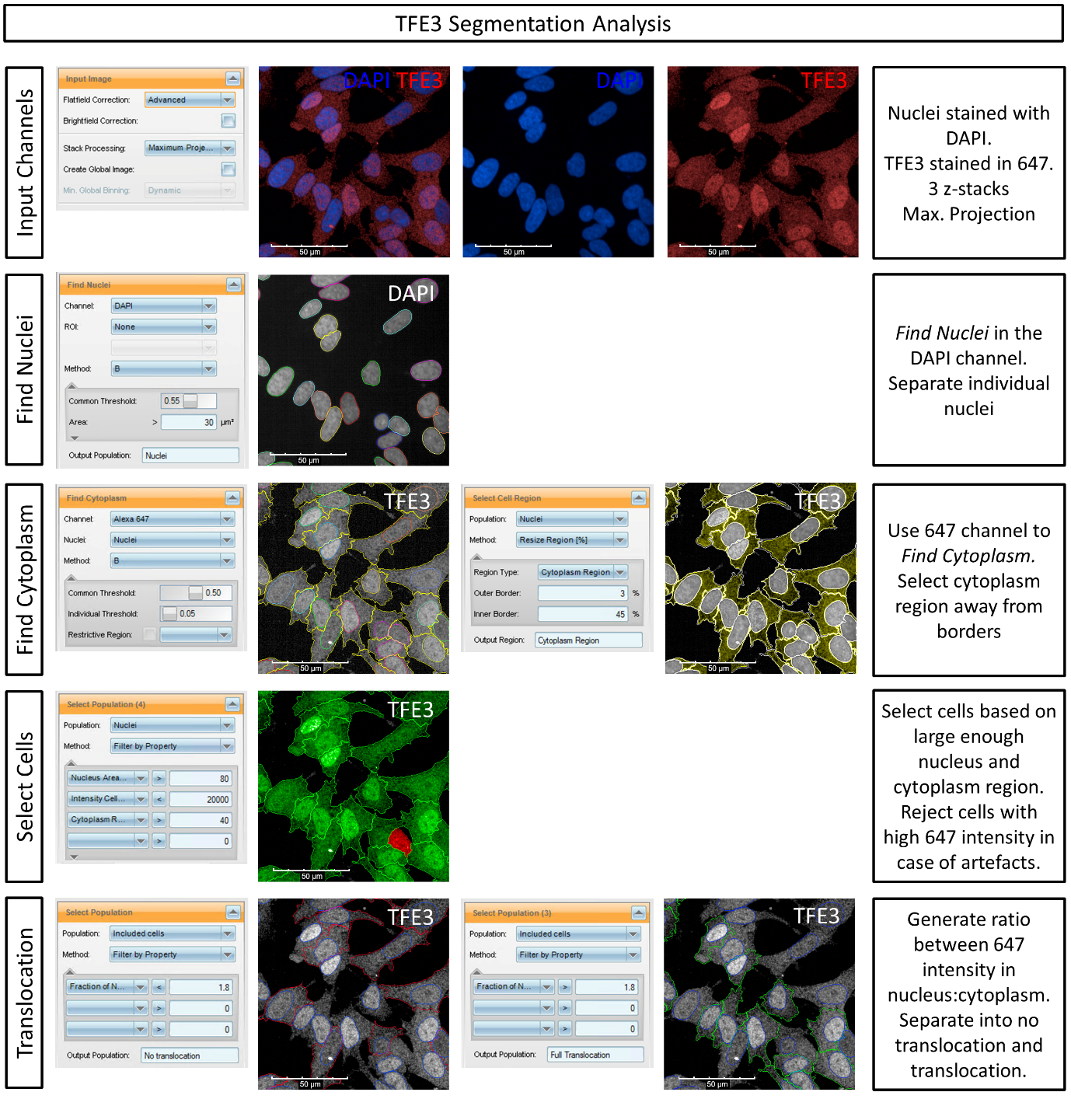


**Figure S3.** TFE3 translocation segmentation analysis. Input images are processed through various image analysis building blocks on Harmony Image Analysis Software. Nuclei and Cytoplasmic Regions are identified. A ratio of the intensity of 647 in nucleus:cytoplasm is calculated and used to distinguish between cells with or without translocation of TFE3. Scale bars: 50 µm.


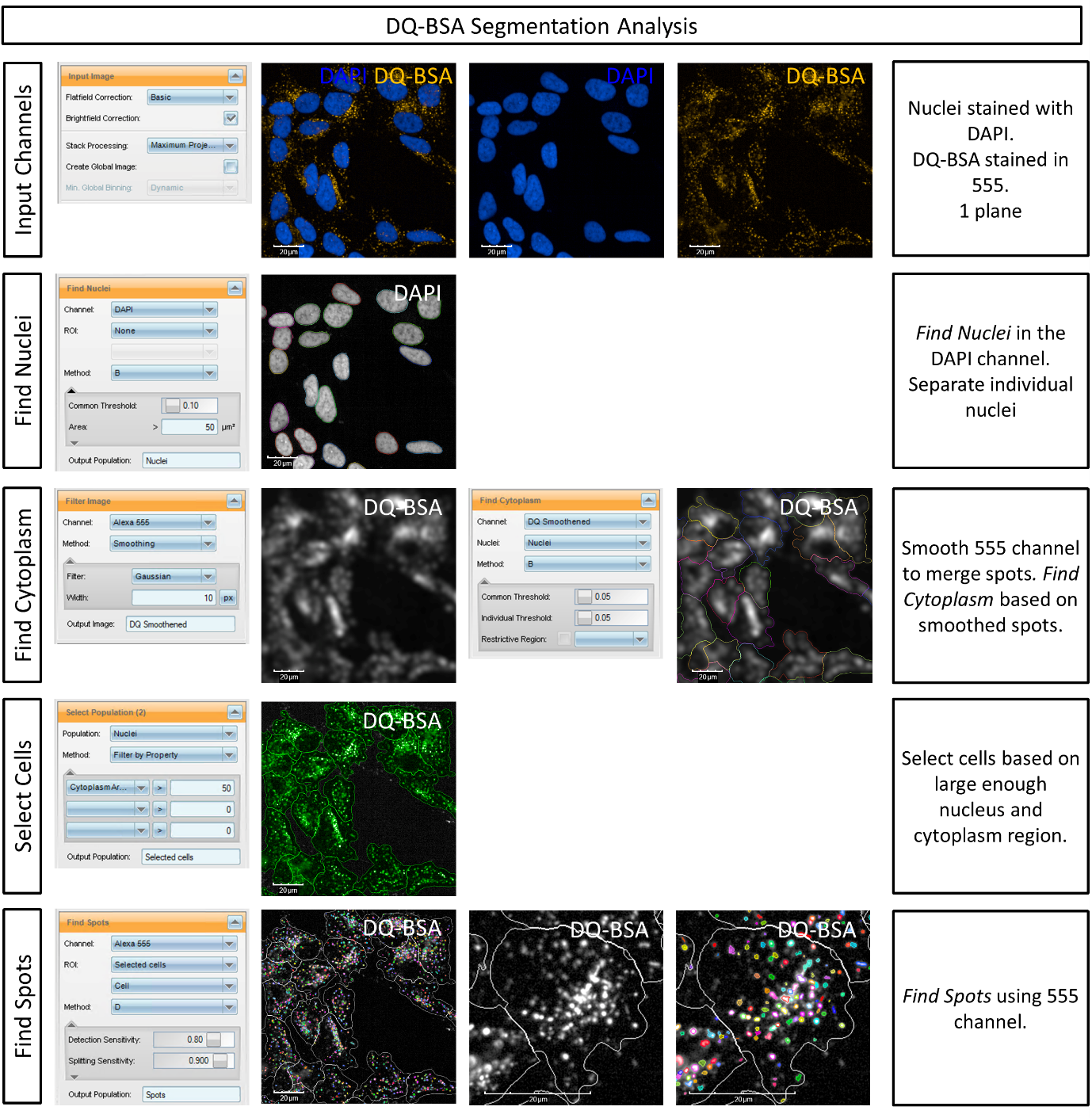


**Figure S4.** Lysosomal DQ Red BSA Spot segmentation analysis. Input images are processed through various image analysis building blocks on Harmony Image Analysis Software. Nuclei and Cytoplasmic Regions are identified. Spots are segmented and counts per cell as well as fluorescence intensity are calculated. Scale bars: 20 µm.


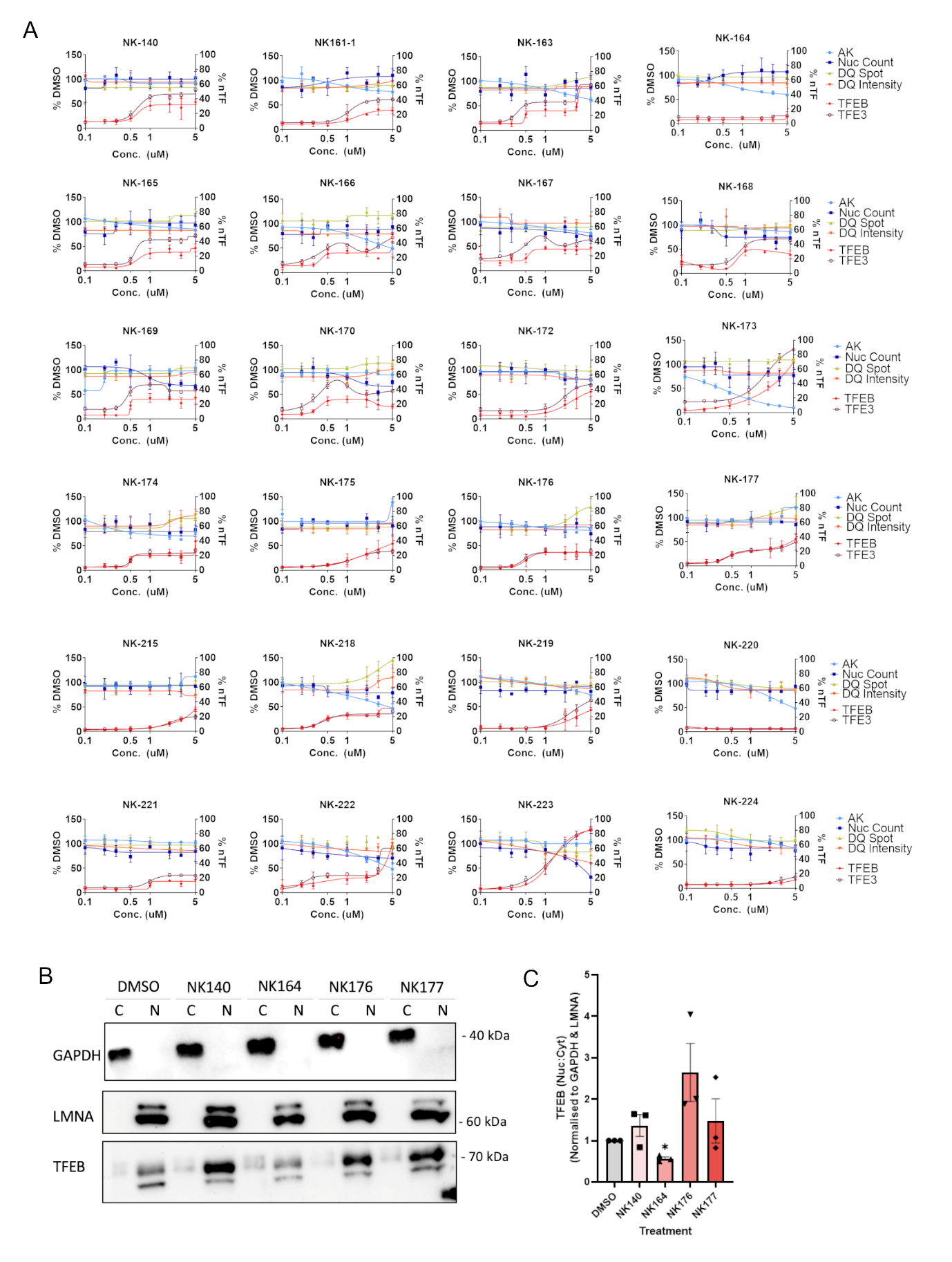


**Figure S5.** Activity of quinazoline analogs. (**A**) The concentration responses across toxicity, lysosomal and TFEB or TFE3 assays for the 24 analogs in a series developed from the original screening hit NK140. Compounds were screen at 24-h treatment time up to 5 µM concentration. Data for each compound is represented on single graphs with their structures shown. n=1 for all above assays, mean±s.d. (**B**) Representative western blot of TFEB levels following treatment of compounds followed by fractionation into cytoplasmic (C) and nuclear (N) compartments. Successful fractionation represented by enrichment of GAPDH in the cytoplasmic fraction and LMNA (lamin A/C) in the nuclear compartment. (**C**) The levels of nuclear/cytoplasmic TFEB were quantified and presented as a ratio. A paired one-way ANOVA with a Holm-Šídák's multiple comparisons test was performed relative to DMSO for comparison between each compound treatments (n=3).


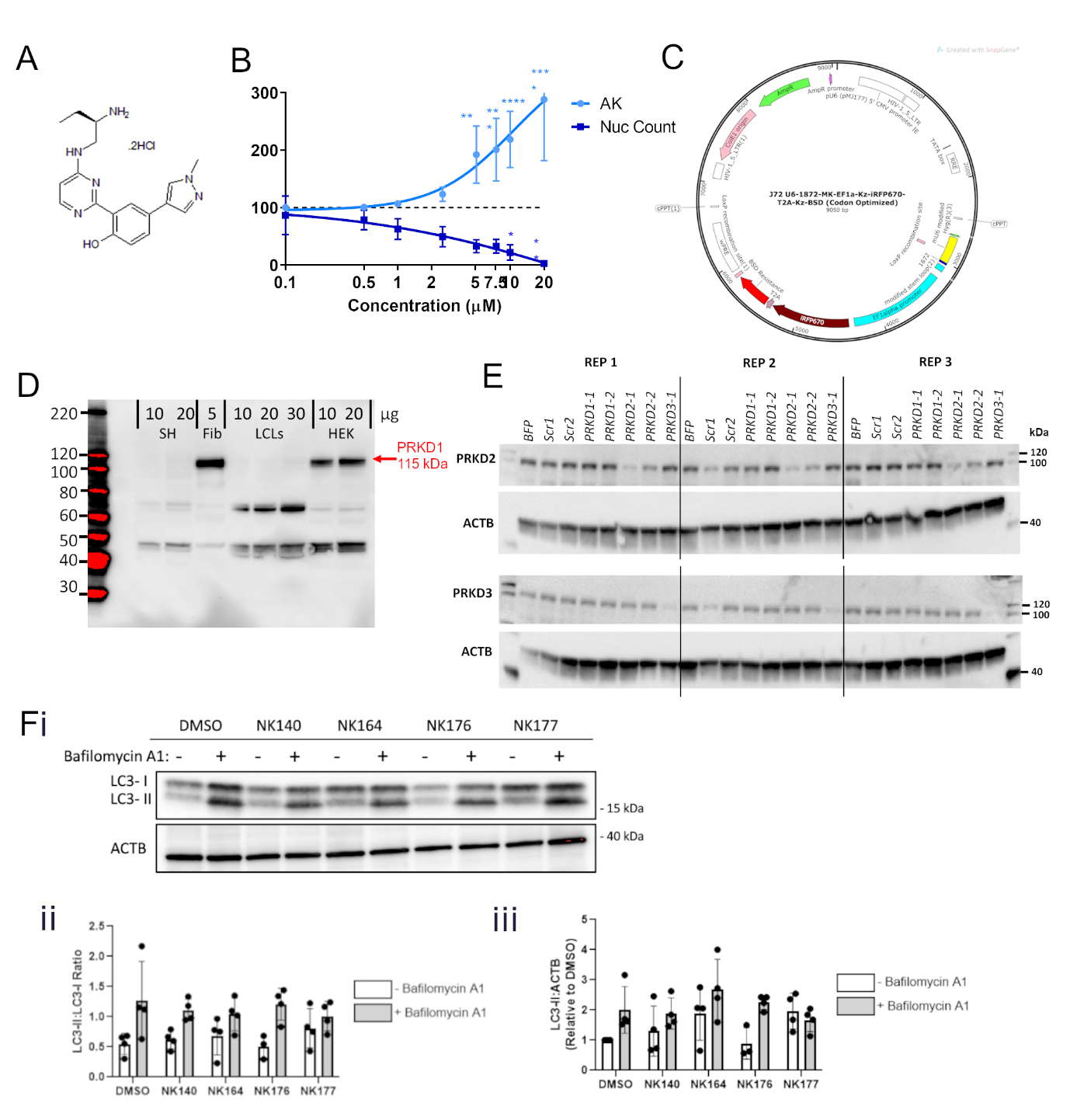


**Figure S6.** PRKD knockdown and inhibition in SH-SY5Y cells and LC3 flux assay. (**A**) CRT0066101 has a diverse chemical structure to other PRKD inhibitors and hit compounds. (**B**) Reduction in nuclear count and increase in AK signal indicates the compound is toxic to cells at concentrations above 1 µM. n=3 for all above assays, mean±s.d. *p<0.05, **p<0.01, *** p <0.001, **** p <0.0001 Two-way ANOVA with Dunnett's multiple comparisons test. (**C**) Vector map for J72, a modified pMK1334 (EF1a-Puro-T2A-2xMYCNLS-WPRE-mU6-sgRNA) plasmid. (**D**) Immunoblotting for PRKD1 shows no detectable band in SH-SY5Y (SH) lysates or lymphoblastoid cell lines (LCLs), despite bands being detectable at the appropriate molecular mass in both fibroblasts (Fib) and human embryonic kidney (HEK) lysates. (**E**) PRKD2 and PRKD3 are detectable at ~120 kDa and expression is measured after using Scr and PRKD1, PRK2 and PRKD3 targeting guides using CRISPRi. (**F**) (i) Representative western blot of LC3 levels following treatment of compounds with or without bafilomycin A_1_. (ii) Quantification of LC3-II:LC3-I ratio. n=3 mean±s.d. A Two-way ANOVA was performed finding a significant effect of bafilomycin A_1_ treatment (**** p<0.0001) but no differences between compound treatments or any interaction (both P>0.05). (iii) Quantification of LC3-II normalized to ACTB. n=3 mean±s.d. A Two-way ANOVA was performed finding a significant effect of bafilomycin A_1_ treatment (** p<0.01) but no differences between compound treatments or any interaction (both P>0.05).
